# On the Urinary Stones and Gravel in Men

**Published:** 1802-01

**Authors:** Cit. Fourcroy


### *On the Urinary Stones and Gravel in Men.* *By* Cit. FOURCROY.

[ Continued from Vol. VI. pp. 527—534. ]

Sect. 3. ON *the claffification of urinary ftones.*—The old claffification of urinary calculi, made according to their figure and their fize, cannot at prefent, where we have acquired fo accurate a knowledge of their internal nature, be retained, as they ought rather to be claffed according to their conftituent particles; however, no regard is to be had to the animal matter, as being found in all urinary concretions, and having no influence on their refpective difference. On comparing paring the refults of the analyfes of more than 600 ftones, I am induced to bring them under three genera; *the firft* of which comprehends fuch ftones as are merely compofed of one fubftance, befides the animal matter; *the fecond* contains urinary concretions, confifting of two ftony fubftances, befides the animal matter; and *the third* comprifes all thofe which are formed by more than three calculous fubftances. Thefe three genera comprehend about 12 fpecies, namely, the firft genus three, the fecond feven, and the third two; all of which I fhall now defcribe; but I muft previoufly remark, that the number of the genera, as well as of the fpecies, is only determined after the obfervations hitherto made, and may confequently be increafed in future.

1. *The firft fpecies* of urinary concretions confifts of lithic acid, and ftones of this kind moft frequently occur, as there were amongft 600, about 150. They are eafily diftinguifhed by their reddifh or high yellow colour, much refembling that of wood, by their brittle, radiant-like, homogeneous, and fine texture, and by their perfect folubility in the lyes of fixed alkalis, without difengaging the fmell of ammonia. Their fize varies from the bignefs of a pea to that of a duck's egg, &c. and their figure is roundifh, fpheroidical, compreffed, oval, oblong, &c. the furface polifhed like marble, but frequently rough and warty; of a crimfon light red, yellowifh, light brown colour, but never white, grey, or black; their ftrata differ in number and thicknefs, and are frequently of a fmooth furface. The fpecific weight of thefe ftones is from 1,276, to 1,786, but generally more than 1,500. The urinary concretions in the kidneys are moftly of this fpecies.

2. *The fecond fpecies* is compofed of lithate of ammonia, and differs from the former by difengaging ammonia on their being diffolved in the lyes of fixed alkalis. Concrements of this kind are generally fmall, of a pale or grey colour, and confift of fine ftrata, eafily feparable from each other; they moftly contain a kernel, which is eafily feparated from the ftrata that cover it. Their figure is generally oblong, compreffed like almonds, and of a fmooth furface, which is frequently cryftalline. Their fpecific weight varies from 1,225 to 1,720. They are entirely foluble in water, particularly when previoufly pulverifed. All acids, principally the muriatic acid, deprive them of the ammonia, leaving the pure lithic acid behind. They are frequently found covered with a thin ftratum of lithic acid. Amongft 600 calculi there were but few of this kind.

3. *The third fpecies*, confifting of oxalat of lime, are eafily to be diftinguifhed by the protuberances and inequality of their furface, whence they have got the appellation of mulberrylike like ſtones; by their hardneſs, grey colour, ſolid texture, their poliſh like ivory, in the inſide, and their particular ſmell on being ſawed, which reſembles that of ſemen. A peculiar characteriſtic, which diſtinguiſhes them from all others, conſiſts in their leaving lime after the calcination, in their being with difficulty ſoluble in acids and not ſoluble in alkalis, and, at laſt, in their being only decompoſed by the lees of carbonats of alkali. They weigh from 1,428 to 1,976, and their ſize varies from that of a calculus renalis to the bigneſs of an egg or more; their figure is generally ſpherical or ſpheroidical. They often make the kernel of other ſtones, in which caſe they belong to another ſpecies. In 300 ſtones they bore the proportion of about $\frac{1}{4}$ or $\frac{1}{5}$.

4. *Stones of this ſpecies* contain lithic acid and phoſphat of earth, but in a ſeparate ſtate. Their ſurface is white, cretaceous, brittle, and half-tranſparent, as it either conſiſts of phoſphat of lime, or of phoſphat of ammoniacal magneſia, the kernel being formed by lithic acid; thus both conſtituents are exactly ſeparate from each other. They were found in the proportion of $\frac{1}{12}$ amongſt the ſtones that were examined by us, and they grow bigger than any of the reſt, as they appear from the ſize of an egg to that of the whole bladder, even when extended. They generally have an oval form, often pointed at one end, of a ſmooth ſurface, which, however, is frequently covered with cryſtals of phoſphat of ammoniacal magneſia. Sometimes the lithic acid in the middle is alternately covered with phoſphat of lime, and phoſpat of ammoniacal magneſia. The ſpecific weight of theſe ſtones is extremely variable.

5. *The fifth ſpecies of calculi* contains, likewiſe, lithic acid and phoſphats of earth, but intimately mixed with each other. Of theſe ſtones a great many varieties are obſerved, depending on the proportionable quantity of their conſtituent particles, as well as on the ſtrata in which they lie above one another. The chief conſtituents, the phoſphats of earth, are ſeparated in different ſtrata, but ſometimes ſo intimately mixed with each other, that it is impoſſible to diſtinguiſh them with the eye; and the analyſis could only ſhew their difference. From this circumſtance ariſe the variety in the colour, figure, and number of the ſtrata. The colour, however, is generally grey, but frequently variegated like marble, ſometimes like ſoap. Their figure is irregular, oval, or globular, and the ſurface moſtly brittle, cretaceous, or whitiſh, as to make us believe that they only conſiſt of phoſphat of lime. The polyedrous ſtones generally belong to this ſpecies, when they have the appearance of being worn away by rubbing. They make about $\frac{1}{5}$ of the

ſtones ftones we have examined. Their fpecific weight varies extremely, the leaft being 1,213, the greateft 1,739.

6. *This fpecies* is conftituted by lithat of ammonia and phofphat of earth, *i. e.* of lime and ammoniacal magnefia; and refembles in its external appearances the fourth fpecies. One of the conftituents, generally the lithat of ammonia, makes the kernel, while a mixture of the two others, but rarely one by itfelf, forms the cruft. Sometimes, however, the kernel contains alfo the phofphats, and the cruft a little lithat of ammonia, which, even in fome varieties, is mixed with pure lithic acid. The ftrata in ftones of this kind are more eafily feparable, and always fmaller than thofe of the fourth fpecies. Their fpecific weight is 1,312 to 1,761; and they are more rarely met with than moft of the reft. Amongft 600 there were only 20 of this kind.

7. Stones of the *feventh fpecies* confift likewife of lithat of ammonia and phofphat of earths, but intimately mixed with each other. They are of a paler colour, much lighter than the fifth fpecies, and difengage a great deal of ammonia on their being treated with kali. We found them only in the proportion of $\frac{1}{40}$ amongft the ftones which we have analyfed. They never grow fo large as the two former.

8. The conftituent particles of the *eighth fpecies* are phofphat of lime and phofphat of ammoniacal magnefia. The pure white colour, the friability, their being infoluble in alkalis, and their eafy folubility even in weak acids, conftitute the chief characteriftics of this fort of ftones, of which we found about 60 amongft 600: Sometimes they are of an enormous fize, of irregular form, rarely round, but frequently of an uneven furface, and refembling an incruftation. Their texture is formed of white brittle ftrata, fometimes interwoven with folid halftranfparent cryftals of ammoniacal magnefia. The crufts formed on foreign bodies that happened to penetrate into the bladder, belong to this fpecies; the fpecific weight of which is 1,138 to 1,473.

9. This fpecies of ftones contain oxalat of lime, but externally uric acid, in more or lefs quantity, and are only to be diftinguifhed by the kernel from the firft fpecies. The proportion of both conftituents, and the fpecific weight, vary extremely, the latter being 1,341 to 1,754. Sometimes the kernel, confifting of oxalat of lime, is only covered on one fide with uric acid, and difcernible on the other by the protuberances with which the furface is variegated; which variety, however, feldom occurs.

10. Stones of this fpecies have, in their centre, oxalat of lime, furrounded by phofphat of earths; the kernel is grey or brown brown and radiant-like, the cruſt white and cretaceous; their ſize and figure differ extremely, and their ſpecific weight is from 1,168 to 1,752. They amount to $\frac{1}{7}$ of the ſtones we have examined.

11. This ſpecies contains ſtones compoſed of three or four calculous ſubſtances, namely, of oxalat of kali, phoſphat of earths, and of uric acid, either pure or combined with ammonia. They rarely occur; and amongſt 600 ſtones we only obſerved ten or twelve. They often conſiſt of three diſtinct ſtrata, viz. In the interior, of oxalat of lime; in the middle, of lithat of ammonia; and the exterior, of phoſphats of earth, which are frequently mixed with uric acid or lithat of ammonia, all which are diſtinguiſhed on their being ſawed through. This ſpecies comprehends three varieties; the firſt of which conſiſts of oxalat of lime, uric acid, and phoſphats of earth; the ſecond contains lithat of ammonia, combined with pure uric acid, and the two other conſtituents; the third has, beſides theſe two ſubſtances, free uric acid and lithat of ammonia, mixed with the phoſphats of earth. We forbear to mention other varieties of this ſpecies, as being leſs remarkable and inſtructive.

12. The laſt ſpecies of calculi is of a very complicated compoſition. The ſiliceous earth ſeems to have taken the place of the oxalat of lime; it is mixed with uric acid and lithat of ammonia, and covered by phoſphats of earth. Stones of this kind are the rareſt of all, and there were only *two* amongſt 600.

Sect. 4. *On the cauſes of the generation of urinary calculi.*— To inquire into the cauſes by which urinary concretions are produced, is both intereſting and uſeful, however attended with the greateſt difficulties. The writings of medical authors are full of conjectures and hypotheſes with regard to this ſubject, on which nothing could be aſcertained before we had acquired an accurate knowledge of the nature of urinary concretions. It is owing to this circumſtance that the moſt enlightened phyſicians acquieſced in aſcribing the immediate cauſe of them to a ſuperabundance of terreous matter in the urine; and Boerhaave, as well as, particularly, Van Swieten, imagined that the urine of all men contained calculous matter in the natural ſtate, and that for the generation of ſtones a kernel was only required, to attract it. That this may be the caſe, in ſome inſtances, is proved by frequent experience; but ſtones produced by foreign bodies, that have accidentally got into the urethra or bladder, are always white and compoſed of phoſphat of earths, and ſeldom or never covered with lithic acid, a ſubſtance which is obſerved to form the ſtones that moſt frequently occur; but even in theſe the kernel conſiſts of a ſubſtance formed in the body body itſelf, as a grain deſcended from the kidneys, &c. which muſt, therefore, have neceſſarily originated in a peculiar internal cauſe. A ſuperabundance of uric acid in ſtony patients, and its more copious generation than in a ſound ſtate, though it ſeems to be one of the principal and moſt certain cauſes, is by no means ſatisfactory, as it only explains the precipitation of ſtony matter from the urine, but not why it unites in ſtrata. A coagulating ſubſtance is required for ſeparating, attracting, and, as it were, agglutinating the condenſible particles that are precipitated. This ſubſtance is undoubtedly the animal matter which we have conſtantly found in all calculous maſſes, and which ſeems to conſtitute the baſis of ſtones, like the membranous gelatina that of bones. It is known that the urine of calculous patients is generally muddy, ductile, in threads, ſlimy, and as if mixed with albumen, which quality it obtains at the moment when the ammonia is diſengaged, or on the addition of kali that ſeparates it from the acid in which it was diſſolved ; and in all caſes of ſuperabundance of lithic acid the urine contains a great quantity of that animal matter, which promotes the precipitation of it, and attracts and unites the particles thus ſeparated. Hence it appears, that every thing capable of increaſing the quantity of that pituitous gluten in the urine, may be conſidered as the remote cauſe of calculous formations. And the old ideas on pituitous temperaments, or ſuperabundant pituita, &c. which were thought to diſpoſe people to a calculous, ſeems to be connected with the late diſcoveries on the nature of urinary ſtones. Though the animal matter appears to be different in different calculi, yet it is certain, that every calculous ſubſtance contains an animal gluten, from which its concrete and ſolid ſtate ariſes ; whence we may fairly ſtate the ſuperabundance of that ſubſtance as the chief and principal cauſe of calculous formation.

There are, however, other cauſes which ſeem to have a particular influence on the nature of urinary ſtones and the ſtrata in which they are formed ; but it is extremely difficult to penetrate and to explain them. We are, for inſtance, entirely ignorant of the manner in which urinary ſtones are formed from the oxalat of lime ; though, from their occuring more frequently in children than in adults, we might be entitled to aſcribe them to a diſpoſition to acor, a cauſe conſidered by Boerhaave as the general ſource of a great number of diſeaſes incident to the infantile age. This opinion ſeems to be proved by the ideas of Bonhomme, phyſician at Avignon, on the oxalic or ſaccharic acid, as the cauſe of mollities oſſium in the rickets ; by this acid being diſcovered in a ſpecies of ſaliva by Brugnatelli ; and, laſtly, by an obſervation of Turgais, who found this acid in the urine of a child diſeaſed with worms. We but rarely obſerve faccharic acid in the human body, which appears to be moftly adventitious, and by which the animal matter is rendered coagulable, and depofited or precipitated with the oxalat of lime; or the oxalic acid decompofes the phofphat of lime, and forms an infoluble combination incapable of being any longer kept diffolved in the urine. It is, however, extremely difficult to determine how far the conftitution of the body is connected with that particular difpofition in the urine, of precipitating fometimes phofphat of lime mixed with oxalat of lime, fometimes phofphat of ammoniacal magnefia, either by itfelf or mixed with lithic acid, &c. &c. Who can explain the reafon, why of 600 ftones there were only two in which filiceous earth could be traced? Still more difficult it is to explain the caufes why the above fubftances precipitate either at once or in different ftrata; but it may fuffice to have fhown how many obfervations and experiments are required, and what accurate attention and perfeverance are neceffary in order to throw light on fo difficult a fubject. We are only enabled to obtain fatisfactory explanations concerning thofe queftions by an accurate chemical analyfis of the urine of calculous patients in different years of their age; an undertaking which, however difficult, has enriched us with fome interefting and fuccefsful refults, which I fhall communicate in the following paragraph:

[ To be continued. ]

